# Modeling of Fiber Orientation‐Dependent R1 Relaxation in Human White Matter In Vivo Within The Framework of The Transient Hydrogen Bond Model

**DOI:** 10.1002/mrm.70371

**Published:** 2026-04-03

**Authors:** Dmitriy A. Yablonskiy, Risto A. Kauppinen, Ekaterina Paasonen, Jeromy Thotland, Mervi Könönen, Pramod Pisharady, Christophe Lenglet, Juhana M. Hakumäki, Olli H. J. Gröhn, Michael Garwood, Alexander L. Sukstanskii

**Affiliations:** ^1^ Mallinckroft Institute of Radiology Washington University School of Medicine St. Louis Missouri USA; ^2^ Department of Electric, Electronic and Mechanical Engineering University of Bristol Bristol UK; ^3^ A.I. Virtanen Institute University of Eastern Finland Kuopio Finland; ^4^ Kuopio University Hospital Neurocenter Kuopio Finland; ^5^ Center for Magnetic Resonance Research University of Minnesota Minneapolis Minnesota USA; ^6^ Department of Clinical Radiology Kuopio University Hospital Kuopio Finland; ^7^ Institute of Clinical Medicine University of Eastern Finland Kuopio Finland

**Keywords:** fiber orientation, magnetic field strength, MRI, R1 relaxation anisotropy, transient hydrogen bond model, white matter

## Abstract

**Purpose:**

Axon fiber orientation‐dependent R1 relaxation in human white matter (WM) in vivo at B0 = 1.5 T, 3 T, and 7 T was studied within the framework of the transient hydrogen bond (THB) model, which attributes MR signal relaxation to quantum magnetization interactions of water and motion‐restricted protons in hydrophilic heads of lipid bilayers forming cellular and myelinated membranes.

**Methods:**

R1 images by MP2RAGE MRI with microstructural DTI and NODDI indices by dMRI from WM were acquired. Angular R1 patterns were experimentally determined in WM with Orientation Dispersion Index (ODI) ranging from 0 to 0.2. The THB model was used to identify the biophysical parameters responsible for the R1 angular and field dependencies in quantitative terms.

**Results:**

At all B0s, non‐monotonic R1 behavior was observed with higher R1 of axons perpendicular to the B0 direction and a broad, low R1 minimum centered around 40° fiber‐to‐field angles. The THB model with the same set of biophysical parameters yielded a good fit for R1 angular patterns in axon fibers oriented between 9.5° and 90° in respect to B0 at all fields. The experimental R1 values in the fibers between the 0° and 9.5° orientations showed an additional dip consistent with their inherent microstructural features. These fibers were found in the cortico‐spinal tract with large and giant axons that would have a lower surface‐to‐volume ratio for forming THB, hence lower R1 relative to tracts with small‐diameter axons.

**Conclusions:**

The data show that the THB model provides a robust physical framework for R1 relaxation anisotropy and B0 field dependence in vivo.

## 1 Introduction

MRI contrast based on T1 relaxation weighting is extensively used in neuroimaging to quantify macrostructural features, such as regional brain volumes, cortical thickness, and shapes of brain structures, collectively known as brain morphometry [1, 2]. Quantitative T1 maps (or R1 maps, R1 = 1/T1) provide information on the number of chemical constituents in brain tissues, such as myelination [3, 4, 5], iron [6, 7], and physical factors, such as temperature [8]. A growing body of evidence suggests that microstructural features in white matter (WM), such as axon fiber orientation with respect to the magnetic field B0, can also influence T1 relaxation [9, 10, 11, 12, 13, 14]. Overall, quantitative T1 images can be considered as unprecedented sources of data from central attributes to brain health and disease.

While T1 relaxation in physical terms is understood as the recovery of the thermal equilibrium of spins, the multitude of factors that influence the relaxation process in vivo make it a complex task to untangle neurobiological correlates to the quantitative T1 values [5]. Different mechanisms, such as water interaction with macromolecular protons and water exchange between tissue compartments, have different time scales and contribute differently to MR signal formation depending on the details of the MRI pulse sequences, so that the numeric values of T1 in cerebral tissue types differ [15]. In addition, the T1 signal in WM has been reported to be multiexponential in several studies [14, 16, 17, 18, 19]. From these perspectives, the measured T1 values should be referred to as apparent. In this regard, it is remarkable that the experimental data obtained using both inversion recovery (IR) [12, 13] and variable flip angle (VFA) [13] pulse sequences have shown consistent angular patterns of T1 (or R1) in WM. Namely, fibers oriented parallel to B0 show a longer T1 relative to those in a perpendicular orientation, and a broad long T1 hump is centered around the 40° fiber‐to‐field angle. Interestingly, the angular patterns show these same features in both WM in vivo [12, 20] and ex vivo [13] and in absolute terms, they measure ˜3%–5% of the mean T1 relaxation time both in vivo [20] and ex vivo [13]. Thus, it appears that the interactions between water and WM microstructural protons result in a T1 (R1) anisotropy contrast that is uniformly observable in quantitative T1 (or R1) images acquired using a range of MRI pulse sequences.

A recently proposed biophysical Transient Hydrogen Bond (THB) model [14, 19] explains the MR signal relaxation in the framework of quantum spin/magnetization exchanges within the THB matrix, encompassing water molecules and protons transiently bound in the hydrophilic heads of cellular and myelin membranes. Importantly, the THB model predicts that the primary source of the anisotropy of tissue T1 relaxation properties is related to the order in the orientations of the water protons—bound protons' transient connections [19]. The THB model has already explained in vivo data on the B0 field dependence of T1 in WM and GM and T1 anisotropy in porcine spinal cord ex vivo [19].

Here, we studied in vivo R1 relaxation angular patterns in human WM at three commonly used magnetic field strengths and analyzed them within the framework of the THB theory. An important feature of our new approach is incorporating into the THB model the B0 field dependence and anisotropy of quantum interactions between bound protons, described in the framework of the Lateral Diffusion Model (LDM [21]). Our results show that the combination of the THB and LDM biophysical models provides a consistent explanation of the R1 anisotropic patterns for all B0 field strengths with the same set of biophysical parameters characterizing quantum interactions within the transient hydrogen bonds.

## 2 Methods

### 2.1 Human Subjects

The study protocols were approved by the Regional Medical Research Ethics Committee (Kuopio, Finland) and the University of Minnesota Institutional Review Board (Minneapolis, MN, USA). Informed consent was obtained from each volunteer prior to enrolment. Eight healthy volunteers (mean age 33 years, range 28–37 years, six females) consented to participate in the study for 1.5 T (Siemens MAGNETOM Sola System, exact Larmor frequency 63.661 MHz) MRI scans at the Kuopio University Hospital. Six healthy volunteers (age range 23–32 years, two females) were recruited at the University of Minnesota, and they were scanned at both 3 T (Siemens MAGNETOM Prisma System, exact Larmor frequency 123.174 MHz) and 7 T (Siemens MAGNETOM AS System, exact Larmor frequency 297.208 MHz) within a period of 6–9 months.

### 2.2 MRI

The details of the MRI scan protocols at all three fields are provided in the Methods section of a recent publication [20]. The MRI acquisition protocols and parameters used are summarized in Supplementary Table 1.

### 2.3 Image Processing

T1 maps were computed from MP2RAGE images by fitting the signals from six inversion time (TI) images into a mono‐exponential model, as described previously [20]. In this regard, it would be accurate to refer to the resulting T1 as ‘apparent T1’ (consequently, ‘apparent R1’), since the values of longitudinal relaxation time (and rate) vary from IR MRI method to another and are also influenced by the acquisition parameters [15]. Hence, the R1 values reported in this study should not be confused with the intrinsic longitudinal relaxation values of each proton pool in WM. dMRI images were processed for FA, MD, and V1 using DTIFIT in FSL and the NODDI procedure [22] to compute the orientation dispersion index (ODI) image from two‐shell dMRI images at all three fields. The FA maps were registered to the R1 (R1 = 1/T1) images with FLIRT in FSL [23], and both V1 and ODI images were then registered on the R1 maps using the transformation matrix from the FA image registrations. Subsequently, the fiber‐to‐field angle (θ_FB_) images were obtained from the V1 and FA images, as described previously [20]. The voxel‐wise R1 relaxation rates in WM were computed in Matlab as a function of $\theta_{\mathrm{FB}}$ and ODI, as described previously [10, 24]. ODI was used as the dMRI microstructural measure to selected WM tissue for R1 angular plots, owing to its ability to limit fiber orientation dispersion within voxels with high FA. The NODDI approach provides more specific measures of WM microstructural complexity than those obtained by FA, owing to its disentangling of neurite density and (fiber) orientation dispersion [22]. An ODI < 0.2, as a measure of WM microstructure, has recently been shown to yield better‐resolved T1 angular features than those obtained with FA > 0.5 alone [24].

### 2.4 THB Model

Our T1 data analysis was based on the THB model [14], which attributes the major features of the water MRI signal relaxation properties in brain tissue to quantum dipole–dipole interactions between water molecules and *subpopulations of protons* in cellular membranes and myelin‐forming lipid bilayers. Specifically, the THB model considers the hydrogen bonding of water molecules from intracellular, extracellular, and myelin water compartments, transiently trapped in hydrophilic heads of cellular membranes and myelin‐forming lipid bilayers, as a biophysical mechanism facilitating these quantum dipole–dipole interactions.

The THB model has two major parameters: *τ*, the THB lifetime, and *λ*, the strength of quantum dipole–dipole spin exchange interactions between water protons that are transiently trapped in lipid heads (w‐protons) and their lipid‐bound counterparts (b‐protons):

(1) $$\lambda =\frac{3}{2}\times \frac{1}{r^{6}}\times {\left(\hbar \gamma^{2}\right)}^{2}$$

where r is the distance between the w‐ and b‐protons in the THB, ℏ is Planck's constant, and γ the gyromagnetic ratio.

It turns out [14, 19], the contribution of THBs with a short *τ* (a few nanoseconds) describes the T1 field dependence fairly accurately, but do not contribute to the T1 anisotropy. THBs with a long *τ* (tens of nanoseconds), instead, contribute mostly to the T1 signal anisotropy, and will be identified below by index “A”. By focusing on the anisotropic properties of T1 signal relaxation, the relevant relaxation rate parameters can be presented as follows:

(2) $$\begin{array}{ll} R_{1}^{w} & =R_{1}^{\mathrm{wI}}+\Delta R_{1}^{\mathrm{wA}},\;\Delta R_{1}^{\mathrm{wA}}=n_{\mathrm{wA}}\cdot \left(k_{A}-n_{\mathrm{bA}}\cdot p_{A}\cdot K_{A}\right)\\ R_{1}^{\mathrm{bA}} & =\left(1-n_{\mathrm{bA}}\right)\cdot r_{1A}+n_{\mathrm{bA}}\cdot k_{A}+n_{\mathrm{wA}}\cdot n_{\mathrm{bA}}\cdot p_{A}\cdot K_{A} \end{array}$$

(3) $$p_{A}=\frac{K_{A}}{\left(1-n_{\mathrm{bA}}\right)\cdot r_{1A}+n_{\mathrm{bA}}\cdot k_{A}-r_{1w}}$$

where $R_{1}^{\mathrm{wI}}$ represents an isotropic component of the water $R_{1}^{w}$ relaxation rate parameter.

The diagonal ($k_{A}$) and cross‐relaxation ($K_{A}$) coefficients are as follows:

(4) $$\begin{array}{ll} k_{A} & =\frac{3}{4}\cdot \lambda_{A}\cdot \left(\frac{1}{9}g_{0}^{A}\cdot L\left(0\right)+2g_{1}^{A}\cdot L\left({\omega \tau }_{A}\right)+g_{2}^{A}\cdot L\left(2{\omega \tau }_{A}\right)\right);\\ K_{A} & =\frac{3}{4}\cdot \lambda_{A}\cdot \left(\frac{1}{9}g_{0}^{A}\cdot L\left(0\right)-g_{2}^{A}\cdot L\left(2{\omega \tau }_{A}\right)\right) \end{array}$$

(5) $$L\left({\omega \tau }_{A}\right)=\frac{\tau_{A}}{1+{\left(\omega \times \tau_{A}\right)}^{2}}$$

where ω=γ×B. The parameters $r_{1w},\;r_{1A}$ represent the contributions to the longitudinal relaxation from the dipole *w‐w* and *bA‐bA* interactions, correspondingly, $n_{\mathrm{bA}}$ is a fraction of *bA* (*b* stands for ‘bound’) protons in the lipid bilayers participating in the *dipole–dipole interaction with water protons* at any given moment, and $n_{\mathrm{wA}}$ is a fraction of water protons interacting with *bA* bound protons at any given moment.

For WM axonal bundles, the coefficients $g^{A}$ are:

(6) $$\begin{array}{ll} g_{0}^{A} & =\frac{1}{8}\times \left(11-30\times \cos^{2}\vartheta +27\times \cos^{4}\vartheta \right),\\ g_{1}^{A} & =\frac{1}{8}\times \left(1+2\times \cos^{2}\vartheta -3\times \cos^{4}\vartheta \right),\\ g_{2}^{A} & =\frac{1}{8}\cdot \left(3+2\cdot \cos^{2}\vartheta +3\cdot \cos^{4}\vartheta \right) \end{array}$$

where ϑ is the angle between the direction of the axonal bundle and magnetic field, B0.

In analyzing the experimental data, we used results for $r_{1A}$ obtained in the framework of the lateral diffusion model (LDM), eq 22 in [21]:

(7) $$\begin{array}{ll} r_{1A} & =\frac{\Lambda }{2}\times \left[\sin^{2}\vartheta \cdot \left(5+3\;\cos2\vartheta \right)\cdot V_{0}\left(\Omega \right)+\left(16\;\cos^{2}\vartheta +6\cdot \sin^{4}\vartheta \right)\right.\\ & \;\cdot V_{0}\left(2\Omega \right)+\left(8-3\;\sin^{4}\vartheta \right)\cdot V_{2}\left(\Omega \right)+\left(8+24\;\sin^{2}\vartheta +3\;\sin^{4}\vartheta \right).\\ & \;\left.\cdot V_{2}\left(2\Omega \right)\right] \end{array}$$

where $\Omega =\omega \times \tau_{d}$, $\tau_{d}$ is the characteristic lateral diffusion time, $\tau_{d}=d^{2}/2D$, where *d* is the minimal “approachable” distance between diffusing protons, *D* is the lateral diffusion coefficient of lipid‐bound protons, and parameter Λ is:

(8) $$\Lambda =\frac{9\pi \cdot n_{2}}{256\cdot D\cdot d^{2}}\times {\left(\hbar \gamma^{2}\right)}^{2}$$

$n_{2}$ is the surface spin density, which can be estimated as $n_{2}=1/d^{2}$, and the functions $V_{0,2}\left(\Omega \right)$ are defined by eq 18 in [21]. While their exact expressions are quite complicated, in the range of Ω values between 5 and 25 (as will be seen below, relevant to our data), the functions $V_{0,2}\left(\Omega \right)$ can be approximated by simple relationships

(9) $$V_{0}\left(\Omega \right)\approx 0.048\cdot \Omega^{-1.10},\;V_{2}\left(\Omega \right)\approx 0.131\cdot \Omega^{-1.31}$$

Since Ω depends linearly on the magnetic field, *B*, we will parameterize it as follows:

(10) $$\Omega =\frac{B}{B_{0}}\cdot \Omega_{0}$$

where $\Omega_{0}=\omega_{0}\times \tau_{d}$ with $\omega_{0}$ corresponding to selected reference field $B_{0}$.

**FIGURE 1:**
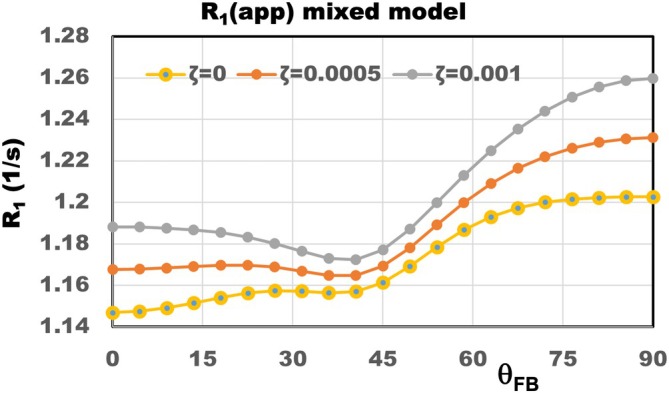
Variation in the R1 anisotropy determined using the mixed model, per Equation (11). The results show that even for a very small mixing parameter ζ (on the order of 0.1%), the characteristics of the R1 angular dependence change from a virtually monotonic increase (at ζ = 0) to a broad minimum at around 40°. (Adopted from [19]).

The total longitudinal relaxation‐related part of the MR signal has contributions from three components: water protons, *bI* (*bI* = bound isotropic) protons of short‐lived THBs that do not contribute to MR signal anisotropy, and *bA* protons of long‐lived THBs contributing to MR signal anisotropy [19]. However, if data are fitted using a single exponential model, the fitting parameter $R_{1}$ (in this case, it should be called an *apparent* $R_{1}$, $R_{1}^{\mathrm{app}}$) actually represents a mixture of $R_{1}^{w}$, anisotropic component $R_{1}^{\mathrm{bA}}$ and isotropic component $R_{1}^{\mathrm{bI}}$. Consequently, we used a mixed‐signal model, where the $R_{1}^{\mathrm{app}}$ can be calculated according to the following equation:

(11) $$R_{1}^{\mathrm{app}}=\left(1-\zeta \right)\cdot \left(R_{1}^{I}+\Delta R_{1}^{\mathrm{wA}}\right)+\zeta \cdot R_{1}^{\mathrm{bA}}$$

where ζ is the mixing parameter, $\Delta R_{1}^{\mathrm{wA}}$ and $R_{1}^{\mathrm{bA}}$ are defined by Equation (2), and $R_{1}^{I}$ is an apparent isotropic component that is mostly defined by $R_{1}^{\mathrm{wI}}$ in Equation (2), with a small mixture from an isotropic *bI* component. The trends in the angular dependencies of $R_{1}^{\mathrm{app}}$ are illustrated in Figure 1 for different values of the mixing parameter ζ.

In the present study, data analysis was based on the Bayesian probability approach developed by Bretthorst and co‐workers [25, 26, 27]. The experimental data were fitted to the theoretical model describing the R1 angular and B‐field dependencies, Equation (11), using a joint analysis for all three magnetic field strengths. In this analysis, all the biophysical parameters characterizing interaction between water and b‐protons ($\lambda_{a},\;\tau_{a},\;n_{\mathrm{wa}},\;n_{\mathrm{ba}},\;\Lambda ,\;\Omega_{0},\;\zeta$) were considered the same for all fields and θ_FB_ angles between 13.5° and 90°, while the parameters $R_{1}^{I}$ were not modeled and were treated independently. The first three points, corresponding to fiber‐to‐field (θ_FB_) angles of 0°, 4.5°, and 9°, were omitted from the fitting procedure, as they fell out of the general trend (see below). A potential cause of such behavior is considered in the Discussion section.

## 3 Results

**FIGURE 2:**
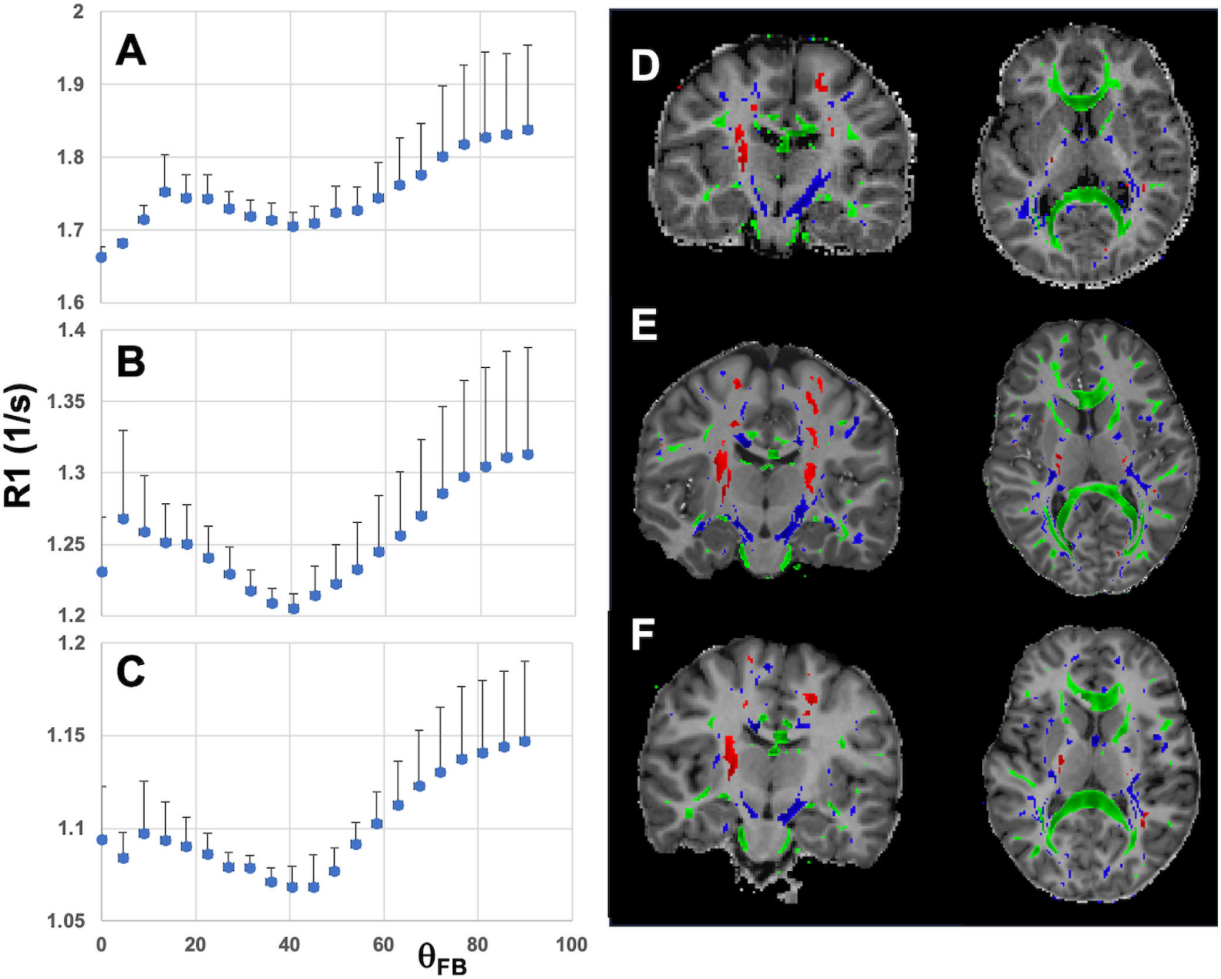
Experimental angular R1 plots from human WM with ODI from 0 to 0.2 at 1.5 T (A), 3 T (B), and 7 T (C). The number of volunteers was eight at 1.5 T, six at 3 T, and six at 7 T. The blue symbols represent the mean values of R1, and the bars represent the SDs. The anatomical distributions of WM voxels (with ODI from 0 to 0.2) are shown for θ_FB_ values from 0°–9.5° (red), 35°–50° (blue), and 75^o^–90° (green) at 1.5 T (D), 3 T (E), and 7 T (F). The distributions of two sets of ODI and θ_FB_ bins are shown superimposed on representative coronal and axial R1 maps of volunteers at each magnetic field strength.

The human WM with ODI from 0 to 0.2 showed the ODI values of 0.12 ± 0.06, 0.13 ± 0.06, and 0.13 ± 0.05 at 1.5 T, 3 T, and 7 T, respectively; the relaxometric data from such tissue were used to compute R1 angular plots. The FA values in such WM were 0.62 ± 0.14, 0.68 ± 0.13, and 0.70 ± 0.13 at 1.5 T, 3 T, and 7 T, respectively. Figure 2A–C displays the R1 plots as a function of θ_FB_ in WM with low ODI at three magnetic fields. The R1 plots show the general trend of perpendicularly oriented axons having higher R1 values than all other orientations. The data also revealed a broad minimum at ˜40° and another dip for parallel or close‐to‐parallel orientations.

**FIGURE 3:**
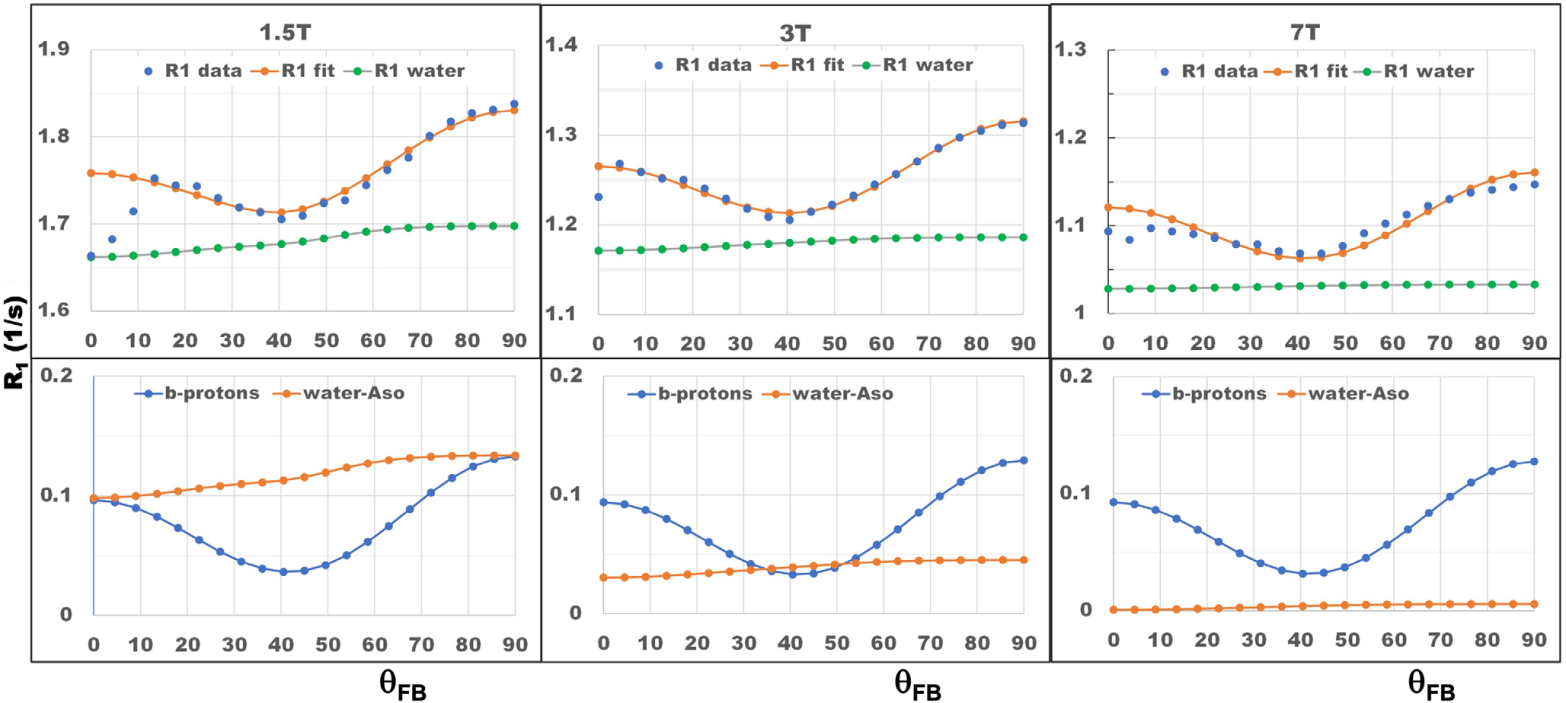
THB model fitting results. Upper panel: Blue dots represent the experimental data corresponding to the mean R1 values in Figure 2A–C. Red dots are the fitting results ($R_{1}^{\mathrm{app}}$) in Equation (11), and green dots show the contribution of the $R_{1}^{w}$ component to the $R_{1}^{\mathrm{app}}$. The corresponding isotropic $R_{1}^{w}$ parts, $R_{1}^{I}$, values are 1.56 s^−1^ for 1.5 T, 1.14 s^−1^ for 3 T, and 1.03 s^−1^ for 7 T. Lower panel: Contribution of *b*‐protons (blue dots) and the anisotropic parts of water signal per Equation (2) (orange dots) to $R_{1}^{\mathrm{app}}$ in Equation (11). The first three points (with θ_FB_ from 0° to 9.5°) were discarded from the model fitting. Note the different ranges of R1 values for different field strengths in the upper panel and between panels.

The results of fitting the experimental data to the theoretical THB model with θ_FB_ > 9.5° (as measured by DTI) are shown in Figure 3, with the fitting parameters listed in Table 1. At all three magnetic fields, this model could explain the general trend of perpendicularly oriented axons having higher R1 than axons in all other orientations and a broad minimum at ˜40°. The theoretical THB model appears thus quite satisfactory for quantitatively describing the R1 angular dependencies for axonal orientations with θ_FB_ > 9.5°, at all three fields using the same set of biophysical parameters ($\lambda_{a},\;\tau_{a},\;n_{\mathrm{wa}},\;n_{\mathrm{ba}},\;\Lambda ,\;\Omega_{0},\;\zeta$) that are specific to tissue microstructure and independent of magnetic field strength. The mean relative fitting error in this interval was 0.35% for 1.5 T, 0.27% for 3 T and slightly higher but still small, 0.83%, for 7 T. The residuals between the fitted and experimental R1 values as a function of θ_FB_ for each B0 are shown in Figure S1. The data in the upper panel of Table 1 show the mean values and uncertainties of the biophysical parameters determined from the Bayesian probability distributions of the model parameters.

**TABLE 1:** The model and derived parameters obtained from the THB model fitting to in vivo experimental R1 angular data from human WM with ODI from 0 to 0.2 at 1.5 T, 3 T, and 7 T.

A	$n_{\mathrm{bA}}$	$n_{\mathrm{wA}}$	Λ [1/s]	Ω_0_ [3 T]	*ζ*
Mean	0.54	0.035	23.1	9.1	0.0025
SD	0.06	0.004	6.2	2.6	0.0003

*B*	*λ*‐Aso [1/s/ns]	*τ*‐Aso [ns]	$\tau_{d}$ [ns]	*d* [Ǻ]	*D* [μm^2^/ms]
Mean	12	70	11.4	2.0	0.0035
SD			3.2	0.1	0.0011

*Note*: Table 1A presents the mean values (upper row) and standard deviations (lower row) of the biophysical parameters determined from Bayesian probability distributions. Proton fractions $n_{\mathrm{bA}}$ and $n_{\mathrm{wA}}$ refer to the fractions of bound and water protons involved in anisotropic *w‐b* interactions in THBs; the parameters Λ and Ω_0_ define the strength and field dependence of *b‐b* interactions in lipid bilayers (see Equations ((7), (8), (9), (10))) and *ζ* is a mixing parameter in Equation (11). The parameters *λ*‐Aso and *τ*‐Aso shown in Table 1B represent the strength and lifetime of THBs. They were fixed during fitting to values obtained in [19] from a spinal cord. The derived parameters, characteristic diffusion time $\tau_{d}$, the minimal “approachable” distance between diffusing protons *d*, and the lateral diffusion coefficient *D* are also shown. The derived parameters were calculated from the fitting parameters using Equation (8) and the definition $\tau_{d}=d^{2}/2D$.

The WM tissue volumes with ODI from 0 to 0.2 were 248 ± 30 mL, 226 ± 25 mL, and 202 ± 24 mL at 1.5 T, 3 T, and 7 T, respectively. The volumes represented 39% ± 2%, 37% ± 2%, and 40% ± 7% of anatomical WM volumes measured in T1 weighted images at 1.5 T, 3 T, and 7 T, respectively. The anatomical distributions of WM voxel bins with close to parallel (θ_FB_ from 0° to 9.5°), intermediate (θ_FB_ from 35° to 50°) and perpendicular (θ_FB_ from 75° to 90°) orientations are shown in Figure 2D–F. WM volumes with θ_FB_ from 0^o^ to 9.5° were 2.3 ± 0.7 mL, 2.4 ± 1.0 mL, and 3.0 ± 1.3 mL at 1.5 T, 3 T, and 7 T, respectively. These volumes corresponded to ˜1.0%–1.4% of WM analyzed for R1 and microstructure, when WM volumes with intermediate orientations (θ_FB_ from 35^o^ to 50°) and close to perpendicular (θ_FB_ from 75^o^ to 90°) ranged from ˜10% to ˜15% and ˜15% to ˜19%, respectively.

## 4 Discussion

We analyzed the effects of interactions involving bound and water protons on the orientation‐dependent R1 relaxation in the framework of the THB model, using MRI data from human WM in vivo acquired at 1.5 T, 3 T, and 7 T. The experimental in vivo R1 angular plots and those computed by the THB model aligned well for WM fibers oriented with $\theta_{\mathrm{FB}}$ from 9.5° to 90° relative to B0 with the same set of biophysical THB model parameters. It is intriguing, but not unexpected, that the R1 values in WM, where axon fibers are at or close to parallel to the B0 field direction, were smaller than could be expected from the THB model with the same biophysical parameters as for θ_FB_ > 9.5° at all three B0s. While the reason(s) for these deviations is(are) not directly evident in these data sets, the observations that the volume of such WM is small and that the tracts with θ_FB_ < 9.5° were found chiefly in the cortico‐spinal tracts (CST) (Figure 2D–F), is informative in this regard. The axon bundles in the CST contain a high number of large/giant axons, as revealed by histology [28] and MRI in vivo [29]. For such axons, the g‐ratio is higher than that of average‐sized axons [28, 30], hence exhibiting thick myelin sheaths in the axons. This leads to a smaller relative surface‐to‐volume ratio for water protons forming THB, and a smaller $n_{\mathrm{wI}}$, defining the isotropic part of R1, and $n_{\mathrm{wA}}$, defining the anisotropic part of R1. A decrease in these parameters consequently leads to a smaller R1 (Equation (2)). Another factor influencing T1 in WM tracts with a high percentage of large or giant axons, is the high proton density relative to the tracts with small axons that results from a high extracellular volume [12, 31]. Indeed, these types of axon fibers are known to have inherently lower R1 (i.e., longer T1) than small axon fibers [12, 31, 32]. Furthermore, it is worth noting that the reported R1 dip at low angles was not evident in the experimental data obtained by the direct rotation of an ex vivo WM sample, as described by Wallstein et al. [13]. This further confirms that the nature of this R1 dip is not related to the axon fiber orientation with respect to the magnetic field, but rather to their large dimensions, resulting in lower $n_{\mathrm{wa}}$ as compared to the axon fibers in WM with θ_FB_ > 9.5°.

The mixed THB model employed in the current data analyses approximates the multi‐component behavior of the MR signal by a single exponential with apparent $R_{1}^{\mathrm{app}}$ in Equation (11). This shows that lipid‐bound protons contribute to the $R_{1}^{\mathrm{app}}$ in two ways—by the direct contribution of the *bA* proton signal $R_{1}^{\mathrm{bA}}$, as described by the second term in Equation (11), and by indirect contribution through water and bound proton interaction in THBs, as per Equation (2). As shown in the lower panel of Figure 3, both contributions to the anisotropic part of $R_{1}^{\mathrm{app}}$, direct and indirect, are of the same order for low B0 (1.5 T), and while the direct contribution remains practically B0‐independent, the indirect contribution dramatically decreases with increasing B0. This suggests that the $R_{1}^{\mathrm{app}}$ anisotropy at high B0 is mostly related to the anisotropy of $R_{1}^{\mathrm{bA}}$ rather than the anisotropy of $R_{1}^{w}$, as also illustrated in Figure 1. This finding is in agreement with results on the orientation dependency of MT [9]. The significance of the direct contribution of *bA* protons to $R_{1}^{\mathrm{app}}$ anisotropy remains important, even though the mixing coefficient *ζ* in Equation (11) is very small, at only 0.0025 (Table 1). The weak B0 dependence of $R_{1}^{\mathrm{bA}}$ is the result of the long (about 70 ns) lifetimes of the *bA* protons, as evident from Equation (5) due to $\omega \times \tau_{A}>>1$. At the same time, the contribution of isotropic interactions, w‐*bI*, depends on B0, as their lifetime is in the range of only a few nanoseconds [14, 21], giving $\omega \times \tau_{A}\approx 1$. These interactions contribute to the isotropic part $R_{1}^{I}$ of the $R_{1}^{\mathrm{app}}$. It is worth noting that previous in vivo studies have also reported the bi‐exponential behavior of the T1 signal [16, 18], but did not address the issue of T1 signal anisotropy.

An important feature of the THB theory is the prediction of the anisotropic behavior of T1 and T2 relaxation. While this theory predicts MR signal dependence due to the interactions between water and bound protons (*w‐bA*), the results also depend on the *bA‐bA* relaxations defined by the relaxation parameters $r_{1A}$ in Equation (2). Herein, the *bA‐bA* relaxation was described in the framework of the LDM model [21], specifically, by the Equations ((7), (8), (9), (10)) above. The angular and B0‐dependencies predicted by the LDM model are essential features of the mixed model, Equation (11), employed in our description of the apparent R1 relaxation, $R_{1}^{\mathrm{app}}$, measured using the MP2RAGE approach.

Our result for the lateral diffusion coefficient of lipid‐bound protons ($3.5\times 10^{-3}{\mathrm{\mu m}}^{2}/\mathrm{ms}$, Table 1) interacting with water protons through THB is well within the range ($10^{-4}-10^{-2}\;{\mathrm{\mu m}}^{2}/\mathrm{ms}$) of previously reported experimental measurements in model bilayer systems for example [18, 33, 34, 35, 36, 37, 38, 39, 40]. In addition, the diffusion‐based component of the spin–spin correlation time, $\tau_{d}$, which we determined to be ˜11 ns, is significantly longer than the rotation correlation times—in the picosecond range [41], making the rotational contribution to the relaxation processes smaller than the diffusion‐mediated interaction between spins. Hence, our data suggest that the lateral diffusion of lipid‐bound protons, rather than their rotations, defines the longitudinal relaxation properties of the in vivo MR signal.

## 5 Conclusions

Our data fits yielded realistic and consistent values for the biophysical parameters of THB interactions at all three B0s, including the fractions of bound and water protons involved in anisotropic interactions and the characteristic features of the lateral diffusion of lipid‐bound protons in the bilayers forming myelin sheaths (such as the lateral diffusion constant and characteristic diffusion time). The R1 behavior of WM tracts oriented < 9.5° with respect to B0 showed an additional dip, most likely related to their specific microstructure. These tracts belonged to the cortico‐spinal WM, where large giant axons are common and require a description with a different set of THB biophysical parameters than axons with orientations > 9.5°. The current observations strongly argue that both the w‐*bA* interactions in the THB and the lateral diffusion of lipid‐bound protons underpin R1 relaxation anisotropy in WM in vivo, thereby providing a solid footing for the biophysical understanding of R1 (or T1) contrast in vivo.

## Funding

This work was supported by Research Council of Finland, #358944. National Institute on Aging, R01AG077658, RF1 AG077658, RF1 AG082030. National Institutes of Health, P30 NS076408, P41 EB027061. National Institute of Biomedical Imaging and Bioengineering, RO3 EB027873.

## Supporting information


**Figure S1:** Residuals between fitted and experimental R1 values at three B0s.
**Table S1:** MRI acquisition parameters for diffusion MRI and MP2RAGE.

## Data Availability

The data that support the findings of this study are available from the corresponding author upon reasonable request.
