# Supplementary material for: Modeling of Fiber Orientation‐Dependent R1 Relaxation in Human White Matter In Vivo Within The Framework of The Transient Hydrogen Bond Model
Source: Magn Reson Med. 2026 Apr 3;96(1):315–22. doi: 10.1002/mrm.70371 (PMC13156443; doi:10.1002/mrm.70371)
Supplement: Supplementary file 1 — Figure S1: Residuals between fitted and experimental R1 values at three B0s. Table S1: MRI acquisition parameters for diffusion MRI and MP2RAGE. [file MRM-96-315-s001.docx]

**SUPPELEMENTARY MATERIAL**

**MODELLING OF FIBRE ORIENTATION-DEPENDENT R1 RELAXATION IN HUMAN WHITE MATTER IN VIVO WITHIN THE FRAMEWORK OF THE TRANSIENT HYDROGEN BOND MODEL**

Dmitriy A. Yablonskiy^1^, Risto A. Kauppinen^2^, Ekaterina Paasonen^3,4^, Jeromy Thotland^5^, Mervi Könönen^6^, Pramod Pisharady^5^, Christophe Lenglet^5^, Juhana M. Hakumäki^5^, Olli H.J. Gröhn^3^ Michael Garwood^5^ and Alexander L. Sukstanskii^1^

^1^Mallinckroft Institute of Radiology, Washington University School of Medicine, St. Louis, MO, USA, ^2^Department of Electric, Electronic and Mechanical Engineering, University of Bristol, Bristol, UK. ^3^A.I.Virtanen Institute, University of Eastern Finland, Kuopio, Finland; ^4^Kuopio University Hospital Neurocenter, Kuopio, Finland. ^5^Center for Magnetic Resonance Research, University of Minnesota, Minneapolis, MN, USA and ^6^Department of Radiology, University Hospital of Kuopio, Kuopio, Finland.

**Figure S1.** Residuals between fitted and experimental R1 values at three B0s.

The residuals between fitted and experimental R1 values (in 1/s) (fitted – experimental) are shown as a function of fiber-to-field-angle (θ_FB_) for 1.5T (blue symbols), 3T (orange symbols) and 7T (grey symbols). The shaded area marks the θ_FB_ range from which the experimental data were not fitted to the THB model.

**Table S1.** MRI acquisition parameters for diffusion MRI and MP2RAGE

| **PARAMETER** | **1.5T dMRI** | **3T dMRI** | **7T dMRI** | **1.5T MP2RAGE** | **3T MP2RAGE** | **7T MP2RAGE** |
| --- | --- | --- | --- | --- | --- | --- |
| Voxel size (mm) | 2.0x2.0x2.0 | 1.5x1.5x1.5 | 1.05x1.05x1.05 | 1.6x1.6x1.6 | 1.2x1.2x1.2 | 0.9x0.9x0.9 |
| Slices | 72 | 92 | 128 | 3D | 3D | 3D |
| TR (ms) | 5500 | 3230 | 7000 | 1650 | 2000 | 3540 |
| TE (ms) | 84.0 | 89.2 | 71.2 | 1.17 | 1.68 | 1.34 |
| MP2RAGE readout pulse and duration | - | - | - | 4^o^ rectangular pulse, 99 μs | 4^o^ slab-selective sinc pulse, 1,000 μs | 4^o^ rectangular pulse, 100 μs |
| GRAPPA | - | - | 3 | 2 | 3 | 3 |
| Phase PF | - | - | - | - | 6/8 | 6/8 |
| Slice PF | - | - | - | - | 6/8 | 6/8 |
| Phase encoding | A>>P, P>>A | A>>P, P>>A | A>>P, P>>A | Linear | Linear | Linear |
| Gradient directions | 20 (AP), 20 (PA) | 197(AP), 197 (PA) | 143(AP), 143(PA) | - | - | - |
| b-values (s/mm^2^) | 750, 1500 | 1500.3000 | 1000,2000 | - | - | - |
| b=0 s/mm^2^ volumes | 2(AP), 2(PA) | 13(AP), 17(PA) | 11(AP), 13(PA) | - | - | - |
| TI (ms) | - | - | - | 170, 250, 600  900, 1200, 1500 | 200,300,600  900,1200,1500 | 300, 600,1000  1500,2000, 3000 |
| Scan duration (min:sec) | 12:16 | 22:38 | 30:48 | 6:06 | 7:45 | 14:03 |

‘PF’ stands for partial Fourier,
